# Solid pseudopapillary tumor of the pancreas: E-cadherin, β-catenin, CD99 new useful markers with characteristic expression (about two case reports)

**DOI:** 10.11604/pamj.2021.40.110.2423

**Published:** 2021-10-19

**Authors:** Nechi Salwa, Dhouib Rym, Doghri Raoudha, Charfi Lamia, Abbes Imen, Driss Maha, Mrad Karima

**Affiliations:** 1 Department of Pathology, Salah Azaiez Institut, Tunis, Tunisia

**Keywords:** Solid pseudo-papillary tumor of the pancreas, CD99, E-cadherin, β-catenin, case report

## Abstract

Solid pseudopapillary neoplasm of the pancreas is a rare tumor that has favorable prognosis. It poses frequently diagnostic challenges. We describe two cases of solid pseudopapillary tumor of the pancreas managed in our department between 2007 and 2011. Two females have mean age of 36.5 years. Clinical presentation include: abdominal pain, bloating and palpable abdominal mass. Tumor is localized in the head of the pancreas in one case and in the tail in the other case. The mean size of the mass was 6 cm (range: 5 to 7 cm). Surgical treatment was performed in two cases. Histological examination confirms the diagnosis of solid pseudopapillary tumor of the pancreas. Immunohistochemical analysis was concordant to the literature data especially concerning CD99 which positivity was in dot, loss of positivity of E-cadherin and nuclear staining of β-catenin. CD10 and α-1-antitrypsin were also positive. One patient was dead 3 days postoperative and neither cancer recurrence nor distant metastases were detected on the follow up of the other. However, solid pseudo-papillary tumor of the pancreas has a distinctive histological appearance; some cases are problematic requiring the use of immunohistochemistry to distinguish it from other pancreatic neoplasm which prognosis is different.

## Introduction

Solid pseudo papillary neoplasm of the pancreas (SPN) is a rare low-grade pancreatic tumor [1, 2]. It was first described by Dr. Frantz in 1959 [2], and was recognized by the World Health Organization (WHO) as “solid pseudo papillary tumors” for the international histological classification of tumors of the exocrine pancreas in 1996 [1, 2]. Its prognosis is generally favorable, in contrast to other pancreatic neoplasms that are characterized by an aggressive behavior. Due to its rarity and unusual behavior, SPN should be differentiated from other pancreatic neoplasms. Despite its characteristic microscopic appearance, the diagnosis of this tumor can be difficult in challenge cases and immunohistochemical analysis can be useful. In this paper, we report two cases of SPN of the pancreas and highlight histological and especially immunohistochemical characteristics of this entity that can differentiate it from other pancreatic neoplasm. CD99, E-cadherin/ß-catenin and CD10 can be considered as relatively specific of SPNs.

## Patient and observation

### Case 1

**Patient information:** a 45-year-old female consults for abdominal discomfort of 6-month duration without jaundice, change in stool color, diarrhea, or flushing.

**Clinical findings:** on physical examination there was mild diffuse abdominal tenderness in all quadrants, as well as a palpable mass on right upper quadrant.

**Figure 1:**
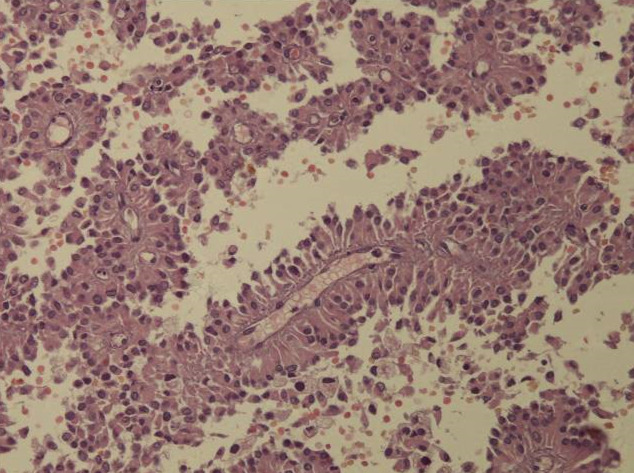
solid pseudopapillary neoplasm; pseudo-papillary component with vessels surrounded by loosely cohesive cells

**Figure 2:**
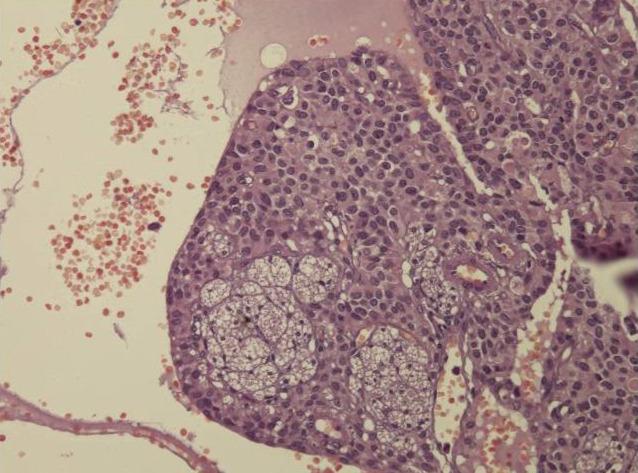
solid pseudopapillary neoplasm; cystic pattern with large hemorrhagic changes and foamy cells

**Figure 3:**
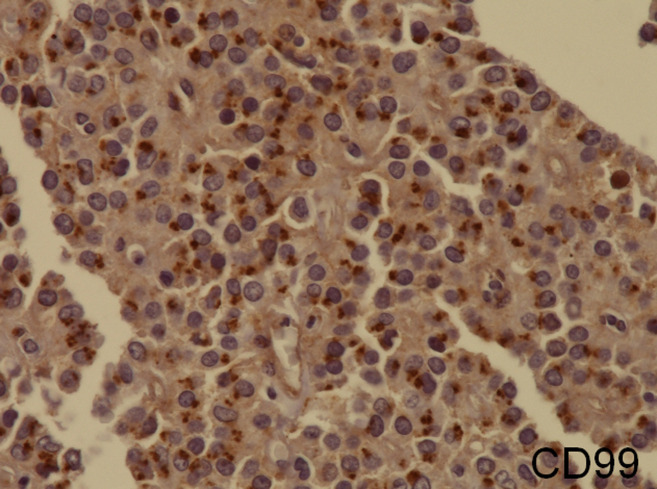
solid pseudopapillary neoplasm; the neoplastic cells show nuclear immunolabelling for β-catenin

**Figure 4:**
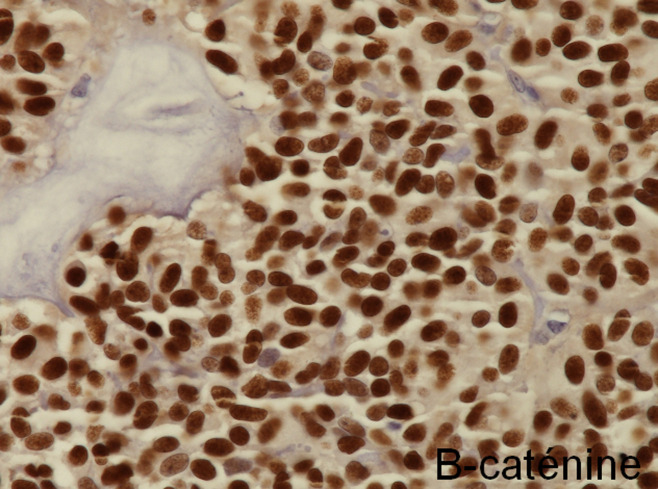
solid pseudopapillary neoplasm; diffuse intracytoplasmic dot-like imunoreactivity for CD99

**Diagnostic assessment:** all blood tests were normal, including tumor markers (CA125, CA19-9). A CT scan revealed a 65 mm, heterogeneous (cystic and solid) mass whose origin (duodenal or pancreatic) cannot be specified. MRI exhibited a 70 mm, solid and cystic tumor at the head of the pancreas evoking a cystadenocarcinoma. At the gross examination of the dudenopancreatectomy specimen, tumor was well limited, encapsulated, solid and cystic, measuring 75/70mm. Cystic component is predominant, containing hemorrhagic material. Solid component was white to grey with hemorrhagic and necrotic degeneration. Microscopic analysis, showed sheets of uniform polygonal cells with a pseudo papillary and cystic appearance (Figure 1, Figure 2). Solid component was made with monomorphic, non-cohesive cells arranged in sheets separated with hyalinized stroma. Neoplastic cells are focally arranged radially around fibrovascular stalks making aspect of “ependymal” rosettes. In immunohistochemestry, tumor cells were positive for β-catenin (Figure 3), CD10, vimentin, CD56, cyclin D1, CD99, progesterone receptors, chromogranin, synaptophysin, cytokeratin. β-catenin positivity was nuclear. Positivity of CD99 was diffuse, in dot (Figure 4). There was loss of expression of E-cadherin.

**Therapeutic intervention:** the patient underwent pancreaticoduodenectomy.

**Follow-up and outcomes:** postoperative period was complicated with pulmonary embolism and patient died 3 days postoperative.

### Case 2

**Patient information:** a 28-year-old female presents with abdominal pain and bloating during 6 months without jaundice or change in stool color or diarrhea.

**Clinical findings:** on examination, there was a diffuse abdominal tenderness.

**Diagnostic assessment:** all blood tests, including tumor markers, were normal. Abdominal ultrasound showed a 50 mm heterogeneous mass in the tail of the pancreas. Abdominal CT scan revealed a cystic mass measuring 50 mm. Macroscopically, there was a firm nodular mass; white to grey with focally cystic degeneration. Histopathology exhibited a proliferation with heterogeneous appearance. It has a papillary architecture (Figure 1, Figure 2) in the center surrounded with massive structures at the periphery. Cells were monomorphous with an eosinophilic, pale cytoplasm and round vesiculous nuclei. There were no mitoses. Stroma was hyalinized. Cells was positive for CD99 with diffuse and in dot expression (Figure 4). They showed nuclear staining with β-catenin (Figure 3). CD10, vimentin, CD56, cyclin D1, progesterone receptors, synaptophysin, cytokeratin were also positive. However, there was loss of expression of E-cadherin and chromogranin was negative.

**Therapeutic intervention:** she underwent distal pancreatectomy with splenectomy.

**Follow-up and outcomes:** patient remains healthy without any sign of recurrence or distant metastasis after 4-year-follow up period.

## Discussion

Solid pseudo papillary neoplasm of the pancreas is a rare tumor accounting for 0.9-2.7% of all exocrine pancreatic tumors, only 5% of cystic neoplasms and 0.2 to 2.7% of the primary non-endocrine tumors of the pancreas [1]. Its origin is still controversial, but it is suggested that it may be derived from the pancreatic duct cells or acinar cells or endocrine cells [2]. It is diagnosed predominately (90%) in young women aged between 25 and 72 years with a mean age of 28 years [1]. Its occurrence in men is rare, and it tends to occur at a more advanced age (mean age: 35 years) [1,3]. The female-male ratio is 5: 1.9 [3]. SPN has no specific clinical symptoms, it is usually found incidentally on routine physical examination or imaging for another indication [2]. It can cause abdominal discomfort, increasing abdominal girth, early satiety, nausea, vomiting and pain [2]. Biological markers are normal and there isn´t a functional endocrine syndrome [4]. Endoscopic ultrasonography and computed tomography are the most frequently used in diagnosing of SPN [2]. They reveal a sharply demarcated, solid and cystic mass without internal septations [2] with enhancement of solid component [2]. Magnetic resonance imaging (MRI) also can be used to diagnosis SPN [2].

Macroscopic examination shows a large, round, solitary mass measuring between 0.5 and 25 cm with an average size of 8-10 cm [1]. It is well-demarcated from the surrounding pancreas and may appear to be encapsulated [5]. The cut section exhibits lobulated, light brown to yellow solid areas and zones of necrosis, hemorrhage and cystic degeneration filled with necrotic debris [1]. Hemorrhagic-cystic changes may be too extensive as to mimic a pseudo-cyst [1]. Microscopically, SPN has a distinctive heterogeneous appearance, with combination of solid, pseudo-papillary and hemorrhagic-necrotic, pseudo-cystic structures in various proportions [6]. Solid areas are composed of poorly cohesive monomorphic cells that are admixed with hyalinized to myxoid stromal bands containing thin-walled blood vessels [1]. They have either eosinophilic or clear vacuolated cytoplasm [1]. Nuclei are round to oval, often grooved or indented with finely dispersed chromatin. Mitoses are rare, however, an important mitotic activity has been observed in some cases [1].

Immunohistochemical findings, as in our cases, showed that SPN has a variable and non specific profile [5]. Neoplastic cells of SPN stain for several markers such as Neuron-specific Enolase (NSE), vimentin, progesterone receptors (PR), a-1-antitrypsin, a-1-antichemotrypsin, CD10, CD56, claudin 5 and 7, galectin 3, cyclin D1 [7]. Inconsistent results have been reported for epithelial markers, synaptophysin, and other antigen such as CEA, CA-19-9 and keratins (7, 8, 18 and 19) [7]. Antibodies to chromogranin A, exocrine enzymes (trypsin, chymotrypsin and lipase), pancreatic hormones, estrogen receptors and a-foetoprotein do not label neoplastic cells [8]. Despite this large number of markers, none seems to be specific and there are still some studies showing that the expression of the above markers overlapped in SPN and other pancreatic tumors. Thus, immunohistochemistry was incapable of being helpful in challenging cases.

Recently, the situation was changed by using E-cadherin, β-catenin and CD99. Many publications reported a loss of E-cadherin and nuclear expression of β-catenin in nearly all cases of SPN [7,9]. In contrast to neuroendocrine Pancreatic Endocrine Neoplasms (PEN), that showed cytoplasm and membrane expression of β-catenin and strong expression of E-cadherin [7]. A study of global gene expression in SPN revealed a gene expression profile that is distinct from that of ductal adenocarcinoma and from PEN [1]. Almost, SPN harbor somatic point mutations in exon 3 of CTNNB1, the gene encoding β-catenin [9]. This mutation leads to β-catenin protein that escapes intracytoplasmic phosphorylation and subsequent and that therefore binds to T-cell transcription factor (Tcf)/lymphoid enhancer binding factor (Lef) [9]. The β-catenin-Tcf/Lef complex is than abnormal translocated to the nucleus, as indicated by the nuclear expression of β-catenin on immunohistochemestry [9]. In the nucleus, the β-catenin-Tcf/Lef complex activates the transcription of several oncogenic genes, among them MYC and cyclin D1 [9]. This leads to activation of the Wnt/β-catenin signaling pathway [9]. Thereby, nuclear expression of β-catenin can be used to differentiate SPN from PEN as reported by Liu *et al*. [7].

CD99 is a 32-KD membrano-cytoplasmic glycoprotein encoded by the MIC2 gene, located on chromosomes X and Y [10]. CD99 functions as an adhesion molecule or as a signal-transduction molecule, and was found to be critical for the regulation of apoptosis [10]. Li *et al*. reported a consistent intracytoplasmic dot-like immunoreactivity of SPN for CD99 [8]. This intracytoplasmic punctuates staining reflects Golgi accentuation of cytoplasmic immunolabelling [8]. However, the specificity of CD99 for SPN is not absolute because it may label non-SPN pancreatic tumors and normal tissue [8]. But, SPN showed a distinctif dot-like cytoplasmic staining. In other tumors commonly reported to express CD99, the staining has been exclusively membranous. Therefore, this data suggest that intracytoplasmic dot-like immune-labelling may be a unique feature of SPN and may be helpful in making the diagnosis. However, the accurate mechanism of this distinct expression of CD99 in SPN is not yet been explained. Although β-catenin, E-cadherin and CD99 are good markers for SPN, no single marker seems to be specific. A core panel of markers may be useful for establishing the diagnosis especially in challenging cases.

## Conclusion

SPN is a rare tumor with non-specific clinical symptoms. Its origin is still unclear. Clinical and radiologic studies are insufficient to establish an accurate diagnosis. Thus tissue sampling and immunohistochemistry are important for diagnosis. However, no maker is specific to establish the diagnosis especially in challenging cases. CD99 combined with E-cadherin/β-catenin and CD10 seem to be helpful. However, further studies are needed to confirm that and to more understand the pathogenesis of SPN.
